# Evaluating the economic burden of dengue in Sri Lanka: A systematic review of costs from 2010 to 2024

**DOI:** 10.1016/j.ijregi.2026.100837

**Published:** 2026-01-07

**Authors:** Hetti T. Wickramasinghe, Randula Ranawaka, Puneet Kalra, Riona Hanley, Elaine Gallagher

**Affiliations:** 1 Department of Paediatrics, Ninewells Hospital, Colombo, Sri Lanka; 2 Department of Paediatrics, Lady Ridgeway Hospital for Children, University of Colombo, Colombo, Sri Lanka; 3 Therapy Area Lead- Vaccines, Takeda Biopharmaceuticals India Private Limited, Gurgaon, India; 4 Takeda Pharmaceuticals International AG, Zürich, Switzerland

**Keywords:** Dengue, Economic burden, Vaccination, Direct cost, Indirect cost

## Abstract

**Objectives:**

This systematic literature review aimed to evaluate the direct and indirect costs borne by households and the health care system in managing dengue cases in Sri Lanka.

**Methods:**

This review evaluates the economic burden of dengue in Sri Lanka from 2010 to 2024, following the Cochrane Handbook for Systematic Reviews of Interventions and PRISMA guidelines. The databases PubMed, Embase, Cochrane, and the Database of Abstracts of Reviews of Effects (DARE) were searched, with a final selection of eight studies that covered direct, indirect, and vector control costs. Costs were converted to 2024 US dollar (USD) using the Sri Lankan Consumer Price Index.

**Results:**

Direct costs ranged from 19 USD per case in southern Sri Lanka to 242 USD nationally, with hospitalization as a major expense. Indirect costs, including lost income from missed workdays, averaged 371 USD per episode, reducing household income by 6.18%. In 2017, the total annual cost of implementing the dengue vaccination strategy was estimated at USD 28.95 million. In comparison, the total cost of a dengue vector control program was USD 400,496, with a cost of USD 2 per person protected over the intervention period (2014-2016). Psychological effects such as post-infectious fatigue, depression, and anxiety were frequently reported.

**Conclusions:**

This review highlights dengue’s economic burden in Sri Lanka, emphasizing the need for cost-effective interventions and mental health support, alongside disease management.

## Highlights

• Eight studies analyzed the cost burden of dengue in Sri Lanka (2010-2024).
• Direct costs ranged from US dollar (USD) 19 to USD 242 per case, mainly due to hospitalization.
• Indirect costs averaged USD 371 per episode, reducing income by over 6%.
• Vector control and vaccination costs reached up to USD 28.95 million annually.
• Psychological impacts such as fatigue, anxiety, and depression were frequently noted.

## Introduction

Dengue virus, transmitted primarily by *Aedes aegypti* and *Aedes albopictus* mosquitoes, has seen a dramatic increase in global incidence over recent decades [1]. The World Health Organization (WHO) identified dengue as one of the top 10 threats to public health worldwide in 2019, with approximately 3.9 billion people in 129 countries at risk of infection [2]. The disease is especially prevalent in tropical and subtropical regions, with 70% of cases occurring in Asia. Sri Lanka is among the top 30 countries with the highest dengue endemicity, with cases continuing to rise despite various control efforts [3,4].

The global economic impact of dengue is substantial, with estimated annual costs of approximately 39 billion USD [5]. Between 2020 and 2050, dengue is projected to impose a burden of 306 billion international dollars (INT$, at constant 2017 prices) on the global economy. Lower-middle-income countries are expected to bear most of both the global health (90.2%) and economic (68.8%) impacts of dengue, with Sri Lanka alone anticipated to experience an economic burden of 1.8 billion INT$ [6].

Sri Lanka, with a population of approximately 23 million and a per capita annual income of 3828 USD, faces significant challenges in managing dengue fever (DF) [7,8]. In 2017, Sri Lanka experienced its largest recorded dengue epidemic, with 186,101 cases and 440 deaths, highlighting the severe impact of this disease on the nation’s public health [8]. As of 2024, the epidemic rate was significantly lower, with 44,880 cases and 22 deaths [9]. The country’s health care system, which had a per capita annual expenditure of 166 USD in 2024 (3.9% of gross domestic product), becomes strained during seasonal dengue epidemics, which coincide with the monsoon rains [10].

Dengue’s rising frequency, severity, and geographic spread amplify health care, prevention, and outbreak management costs in Sri Lanka, impacting patients, households, and the state-funded public health care system. During epidemics, the inability to predict which patients will develop severe disease sometimes results in unwarranted hospitalizations, placing an additional burden on the health care system [11]. The estimated average cost of hospitalization for patients with dengue in Sri Lanka ranges from 239 to 1056 USD for adults and 264-743 USD for pediatric patients, with personnel costs comprising nearly half of these expenses [12]. Understanding the economic burden of dengue is essential for informing policy, prioritizing resources, and guiding interventions. Economic burden data enable assessment of control strategies and aid in targeting effective solutions [13,14].

This systematic review aims to comprehensively assess the economic burden of dengue in Sri Lanka from 2010 to 2024. Its objectives are to evaluate the direct medical and non-medical costs borne by patients and the health care system, quantify indirect costs such as productivity loss and household income reduction, and analyze the financial implications of vector control and preventive interventions, including vaccination programs. The novelty of this study lies in its integration of data from national, regional, and household-level studies, adjusted to 2024 USD for comparability. By consolidating over a decade of evidence, this review provides the first comprehensive, inflation-adjusted estimate of dengue’s economic burden in Sri Lanka, offering critical insights to support policymakers in developing cost-effective prevention and control strategies.

## Methods

The systematic literature review (SLR) was conducted in accordance with the Cochrane Handbook for Systematic Reviews of Interventions and the PRISMA (Preferred Reporting Items for Systematic Reviews and Meta-Analyses) guidelines [15]. The study was registered with PROSPERO, under the registration number CRD42021266800.

### Data sources and search strategy

To identify articles on the economic burden of dengue in Sri Lanka, the databases PubMed, Embase, Cochrane Reviews, Cochrane CENTRAL, and Database of Abstracts of Reviews of Effects (DARE) were searched. The search strategy involved MeSH (Medical Subject Headings), Emtree, and free-text terms, focusing on English articles from 2010 to 2024. The keywords used were dengue, economic impact, Sri Lanka, cost of dengue, direct cost, and indirect cost. Additionally, gray literature searches were conducted across the websites of government and public health agencies and major universities in Sri Lanka, including the WHO Library Information System (WHOLIS), Sri Lanka Ministry of Health, WHO South-East Asia Regional Office (SEARO), Western Pacific Surveillance and Response (WPSAR), and ReliefWeb. Bibliographies of selected articles were also reviewed for additional studies.

### Eligibility criteria and study selection

Articles from databases and gray literature were screened using a modified PICOS (population, intervention, comparator, outcomes, and study design) framework and predefined criteria, with study selection in two phases and a PRISMA diagram illustrating the flow of included and excluded studies. Studies were selected for inclusion in the SLR following a two-stage process: the first stage involved two independent reviewers, with conflicts resolved through discussion, and the second stage involved a single independent reviewer. Exclusion criteria included studies not reporting on patients with dengue or previous exposure to dengue and studies conducted or reporting data outside of Sri Lanka.

### Data extraction, risk of bias assessment, and data analysis

Key data from the selected studies were collected using a data extraction form (DEF) and subsequently synthesized descriptively, supplemented with tables and figures where feasible. Data extraction and risk of bias (RoB) assessment were validated by a second reviewer for 62% of all articles. Discrepancies between the reviewers were resolved through consensus or by a third reviewer. Any required changes to the DEFs were implemented in the second phase, and the remaining eligible studies were subsequently extracted. Where possible, costs were adjusted and converted to 2024 USD using the Sri Lankan Consumer Price Index.

For the RoB assessment, the NHS (National Health Service) Wales tool was used to assess all included cost-of-illness studies. RoB was assessed for all publications, and the complete assessments are summarized in the respective DEFs [16].

## Results

### Study selection and included studies

**Figure 1:**
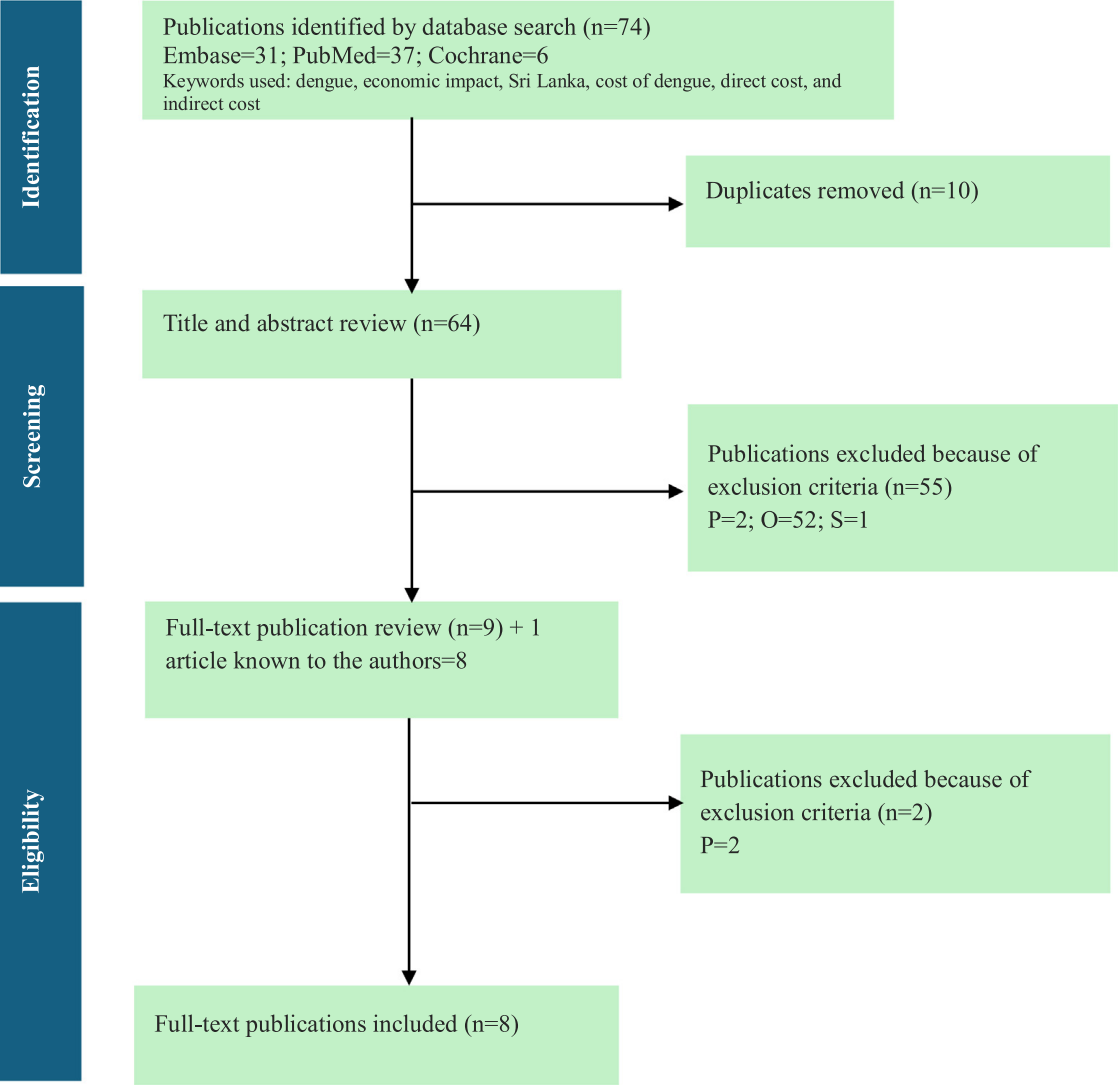
PRISMA flowchart of the cost study selection process. D, duplicates; O, outcome; P, population; S, study design. The PRISMA diagram visually represents the flow of information through different phases of systemic review, it maps out the number of literatures identified, included, and excluded.

A total of 74 cost-related publications were identified through comprehensive literature searches, with 31 from Embase, 37 from PubMed, and six from the Cochrane Database of Systematic Reviews and Cochrane CENTRAL. After removing 12 duplicate records, 62 unique publications remained for screening. The titles and abstracts of these publications were carefully reviewed, leading to the selection of nine publications for full-text review. In addition, one article identified by the authors was included in the study [17]. Following the full-text assessment, eight studies were deemed suitable for data extraction and inclusion in the SLR. The detailed process of study selection is illustrated in the PRISMA diagram (Figure 1).

### Characteristics of included economic burden studies

The characteristics of the studies included in the cost analysis of this SLR are summarized in Table 1. The studies comprised two economic evaluations, four observational studies, one retrospective cost analysis, and one systematic analysis. Of these eight studies, two provided national-level dengue cost data, five offered district- or province-level data, and one study presented both national- and district-level data [7,10,11,14,[18], [19], [20], [21]]. The economic analyses in four studies were conducted from the health care provider’s perspective, while one study took a societal perspective. Three studies did not specify the perspective of the analysis.

**Table 1:** General characteristics of cost studies included in the systematic literature review.

Author, year	Study design	Location	Perspective	Year of valuation and currency	Setting	Direct medical costs	Direct non-medical costs	Total direct costs	Indirect costs	Vector control costs	Surveillance costs	Overall costs
Weerasinghe et al. [11 p11]	Prospective cohort study	Southern province	Societal	2018; USD	Hospitalized dengue	Yes	Yes	Yes	Yes	No	No	Yes
Sonali Fernando et al. [18 p20]	Prospective cohort study	Southern province	Societal	2013; USD	Hospitalized dengue	Yes	Yes	Yes	Yes	No	No	Yes
Sigera et al. [19 p19]	Prospective cohort study	Southern province	Health care	2018; USD	Hospitalized dengue	Yes	No	Yes	No	No	No	No
Tissera et al. [8 p8]	Retrospective observational study	National	NR	2017; USD	NR	No	No	No	No	Yes	No	No
Liyanage et al. [20 p18]	Economic evaluation	Panadura, Kalutara	NR	2016; USD	Hospital-based dengue notification	No	No	No	No	Yes	No	No
Perera et al. [21 p21]	Economic evaluation	National and districts	NR	2017; USD	NR	No	No	No	No	Yes	No	Yes^a^
Thalagala et al. [12 p12]	Retrospective cost analysis	Both	Societal	2012; USD	Hospitalized (dengue ward, intensive care unit)	Yes	Yes	Yes	No	Yes	No	No
Shepard et al. [17 p17]	Systematic analysis	National	Societal^c^	2013; USD	Ambulatory and hospitalized	Yes	Yes	Yes	Yes	No	No	Yes^b^

NR, not reported; USD, US dollar.

a Costs represent the total annual cost of vaccination plus screening.

b Costs represent direct and indirect cost of dengue.

c Societal perspective is assumed since direct and indirect costs were reported.

Direct costs, categorized into medical and non-medical/hospital stay costs, were reported in five studies, while four studies reported on costs related to vector control. Specifically, four studies reported the costs of dengue in hospital settings, and one study provided data on ambulatory care costs.

### Costs due to dengue

#### Direct costs

A retrospective analysis of public sector costs related to dengue control activities and hospitalization costs in the Colombo district during the 2012 dengue epidemic reported the overall hospitalization cost in the Colombo district to be 4,356,343 USD, with medical costs and hospital personnel costs contributing 34% and 46% of the total, respectively. The study further detailed that the average medical cost per patient in the dengue ward was higher for pediatric patients (≤12 years) than adults (>12 years), with costs of 89 USD and 226 USD for DF and dengue hemorrhagic fever (DHF) in children, and 56 USD and 160 USD for DF and DHF in adults, respectively. The cost differences were more pronounced in intensive care unit settings, where the average treatment cost per adult was significantly higher than that for children (Table 2) [12].

**Table 2:** Direct and indirect costs of dengue in Sri Lanka.

Author, year	Region	Population	Setting	Costs (USD)
Weerasinghe et al. [11 p11]	Southern Sri Lanka	Hospitalized dengue	Medical visit cost	10
			Medication cost	5
			Cost of investigations	12
			Travel for medical care	14
			Overall direct non-medical cost	14
			Overall direct cost	21
Sonali Fernando et al. [18 p20]	Southern Sri Lanka	Hospitalized dengue		
		DF Pediatric	Direct medical cost per episode	12
		DHF/DSS Pediatric	Direct medical cost per episode	15
		DF Pediatric	Direct non-medical cost per episode	42
		DHF/DSS Pediatric	Direct non-medical cost per episode	60
		DF Pediatric	Indirect cost per episode	15
		DHF/DSS Pediatric	Indirect cost per episode	33
		DF Pediatric	Total cost per episode	71
		DHF/DSS Pediatric	Total cost per episode	106
Sigera et al. [19 p19]	Western Sri Lanka	Hospitalized Dengue Cases		
		All patients	Total cost	22
		Adults ≤ 20 years	Total cost	22
		Adults 21-30 years	Total cost	22
		Adults 31-40 years	Total cost	22
		Adults 41-50 years	Total cost	22
		Adults 51-60 years	Total cost	23
		Adults 61-70 years	Total cost	21
		Adults ≥ 71 years	Total cost	24
Shepard et al. [17 p17]	National	Total: 688,294 Hospitalized: 120,776 Ambulatory: 392,827 Non-medical: 174,701 Deaths, children: 73 Deaths, adults: 218	Direct cost per non-fatal case	
			Hospital cases	504
			Ambulatory cases	84
			Non-medical cases	10
			Indirect cost per non-fatal case	
			Hospital cases	85
			Ambulatory cases	48
			Non-medical cases	48
			Dengue deaths cost/case	
			Child	155,914
			Adults	101,754
			Annual direct costs	
			Hospital cases	60,871,592
			Ambulatory cases	32,565,312
			Non-medical cases	1,648,588
			Aggregate costs	95,085,494
			Annual indirect costs	
			Hospital cases	10,403,652
			Ambulatory cases	18,962,892
			Non-medical cases	8,433,291
			Fatal cases	33,477,923
			Aggregate costs	71,277,781
			Total annual cost by treatment setting	
			Hospital cases	71,275,243
			Ambulatory cases	51,528,204
			Non-medical cases	10,081,904
			Fatal cases	33,477,923
			Aggregate costs	40,831,912
			Cost of dengue per case	242
Thalagala et al. [12 p12]	Colombo District	Average cost of dengue per patient by setting in 2012		
		DF Pediatric (≤12 years)	Medical cost (ward)	89
		DF Pediatric (≤12 years)	Medical cost (ICU)	139
		DHF Pediatric (≤12 years)	Medical cost (ward)	226
		DHF Pediatric (≤12 years)	Medical cost (ICU)	781
		DF Adults (>12 years)	Medical cost (ward)	56
		DF Adults (>12 years)	Medical cost (ICU)	579
		DHF Adults (>12 years)	Medical cost (ward)	160
		DHF Adults (>12 years)	Medical cost (ICU)	1232
		DF Pediatric (≤12 years)	Hospital stay cost (ward)	288
		DF Pediatric (≤12 years)	Hospital stay cost (ICU)	288
		DHF Pediatric (≤12 years)	Hospital stay cost (ward)	288
		DHF Pediatric (≤12 years)	Hospital stay cost (ICU)	288
		DF Adults (>12 years)	Hospital stay cost (ward)	288
		DF Adults (>12 years)	Hospital stay cost (ICU)	288
		DHF Adults (>12 years)	Hospital stay cost (ward)	288
		DHF Adults (>12 years)	Hospital stay cost (ICU)	288
		DF Pediatric (≤12 years)	Hospitalization cost (ward)	377
		DF Pediatric (≤12 years)	Hospitalization cost (ICU)	427
		DHF Pediatric (≤12 years)	Hospitalization cost (ward)	515
		DHF Pediatric (≤12 years)	Hospitalization cost (ICU)	1068
		DF Adults (>12 years)	Hospitalization cost (ward)	344
		DF Adults (>12 years)	Hospitalization cost (ICU)	867
		DHF Adults (>12 years)	Hospitalization cost (ward)	448
		DHF Adults (>12 years)	Hospitalization cost (ICU)	1520
		DF Pediatric (≤12 years)	Total cost of dengue hospitalizations in 2012 by setting (ward)	1,407,472
		DF Pediatric (≤12 years)	Total cost of dengue hospitalizations in 2012 by setting (ICU)	3411
		DHF Pediatric (≤12 years)	Total cost of dengue hospitalizations in 2012 by setting (ward)	1,412,213
		DHF Pediatric (≤12 years)	Total cost of dengue hospitalizations in 2012 by setting (ICU)	132,499
		DF Adults (>12 years)	Total cost of dengue hospitalizations in 2012 by setting (ward)	517,223
		DF Adults (>12 years)	Total cost of dengue hospitalizations in 2012 by setting (ICU)	5201
		DHF Adults (>12 years)	Total cost of dengue hospitalizations in 2012 by setting (ward)	835,778
		DHF Adults (>12 years)	Total cost of dengue hospitalizations in 2012 by setting (ICU)	42,546
		Overall cost of dengue hospitalizations in 2012		
		Overall population	Medical cost	1,474,303
		DF Pediatric (≤12 years)		335,062
		DHF Pediatric (≤12 years)		718,578
		DF Adults (>12 years)		87,919
		DHF Adults (>12 years)		332,746
		-	Hospital personnel	1,985,593
		-	Hospital utilities, operational and maintenance	895,822
		-	Total, overall cost	4,356,343

DF, dengue fever; DHF, dengue hemorrhagic fever; DSS, dengue shock syndrome; ICU, intensive care unit, USD, US dollar.

Note: The Sri Lankan rupees were first converted to 2017 USD using the exchange rate of 152.5 USD reported in Tissera et al. [8] before inflating to 2024 USD using the Sri Lanka Consumer Price Index.

The economic burden of symptomatic dengue was estimated using 2013 incidence data in Sri Lanka, reporting an overall average cost of 242 USD per case. Costs for hospitalization, ambulatory visits, and non-medical care were reported as 504 USD, 84 USD, and 10 USD per case, respectively. After adjusting for underreporting, the total annual cost of dengue was estimated at 166.36 million USD, with 95.09 million USD attributed to direct costs [17].

A prospective cohort study conducted at a tertiary and secondary-care public hospital from June to October 2017 evaluated the costs associated with illness for dengue hospitalized patients in southern Sri Lanka. Overall, a total median cost of 17 USD was reported, of which 13 USD was accumulated before admission and 7 USD after discharge [11].

#### Indirect costs

Shepard et al. also reported that indirect costs were substantially higher than direct non-medical costs (48 USD vs. 10 USD per case, respectively). The study highlighted that the cost per dengue-related death was higher in children than in adults, despite a lower number of deaths among children. The total annual aggregated indirect cost of dengue was 71.28 million USD, with fatal cases accounting for almost half of this amount [17].

Weerasinghe et al. [11] reported that the mean income loss for households due to hospitalized adult patients and their caregivers was 369 USD and 50 USD in the years 2017 and 2018, respectively. Overall, the total mean income loss attributable to missed workdays during a dengue episode was 382 USD, which resulted in a 6.18% reduction in annual household income.

#### Vector control and surveillance costs

An economic evaluation (Markov model) conducted to determine the cost-effectiveness and budget impact of a hypothetical dengue vaccination strategy combined with a mandatory serological pre-vaccination screening in children aged 9 years in Sri Lanka estimated the total annual cost of vaccination plus screening. The cost perspective was not reported in the study, although data input in the model included both direct and indirect (out-of-pocket and loss of income) costs of dengue. At the national level, the total annual cost of the dengue vaccination strategy in 2017 was estimated at 28.95 million USD, with Colombo and Gampaha contributing the highest costs (3.28 million USD and 3.26 million USD, respectively), consistent with the national incidence. Other districts contributing over 1 million USD each included Kandy (1.96 million USD), Ratnapura (1.56 million USD), Kalutara (1.72 million USD), Kurunegala (2.31 million USD), and Galle (1.50 million USD). The total vaccination cost was estimated at 100 USD per case (vaccine 73 USD and screening 27 USD) [21].

The cost-effectiveness of the dengue vector control program (the Civil-Military Cooperation [CIMIC]) in Panadura, Kalutara District, from June 2014 to December 2016, was evaluated from the perspective of the national dengue control unit and included the direct costs of planning and implementing the CIMIC program and treatment costs (ambulatory and hospitalization). During the study period, the total cost of the dengue control program was estimated at 400,496 USD, and the cost per person protected over the intervention period was 2 USD [20].

The overall cost of dengue control activities in the Colombo district in 2012 was 1,704,332 USD, mainly driven by personnel cost (79%) and consumables (16%), including larvicides, insecticides, kerosene oil, chemicals, petrol, and diesel [12].

From the perspective of the Ministry of Health, the nationwide total direct cost of dengue control and outbreak-response activities was 17.44 million USD, of which 65% was due to the total cost of routine dengue control activities (11.34 million USD), and 35% was due to the total cost of dengue outbreak response (5.98 million USD) [8].

#### Productive days lost

Across the analyzed studies, the average number of workdays missed by adult patients between 2017 and 2018 was 21.5 days, while children lost an average of 13.8 school days [10]. A study focusing on the pediatric population found that patients missed an average of 4.6 school days, while caregivers lost an average of 4.5 workdays during the illness period. From hospital discharge to full recovery, patients missed an additional 7.2 school days, and caregivers lost 3.6 workdays on average [18].

#### Psychological burden of dengue

Three studies across Sri Lanka reported on the psychological burden associated with dengue. In a retrospective case–control study, 15.1% of patients who had dengue 6 to 12 months before their enrollment in the study were also diagnosed with depression, compared with 7.5% of matched controls, highlighting a notable increase in post-infectious depressive symptoms [22]. In another study, a longitudinal analysis was conducted for 480 patients in Colombo from July 2018 to January 2019. The study reported a high prevalence of post-infectious fatigue (35%) at 1 month following dengue infection, along with poor sleep quality, headaches, and persistent myalgia [23]. A 2017 study revealed that 41.4% of patients with dengue experienced anxiety, while 42.5% reported symptoms of depression, further underscoring the significant psychological impact of dengue [24].

#### RoB assessment for economic studies

The RoB of five of the identified studies was assessed using the NHS Wales RoB checklist. Based on the assessment of each question on the checklist, a total rating of good (>70% score), fair (50-70% score), or poor (<50% score) quality was assigned to each study. Overall, three studies were rated good, and two studies were rated fair [8,12,17,20,21]. Potential sources of bias include the lack of comparison of the study results across different subpopulations, settings, and sectors (n = 3 studies); lack of reporting of a range of estimates under different scenarios to ascertain the certainty of the case base analysis; and the reliability of the average estimates [8,20,21].

## Discussion and conclusion

This systematic review provides a detailed evaluation of the economic burden of dengue in Sri Lanka, synthesizing data from studies conducted across various regions and levels of the health care system. The review identifies hospitalization, treatment costs, lost productivity, and prevention efforts as the primary cost drivers of dengue management.

The cost of dengue management varies significantly across studies and regions. Thalagala et al. [12] reported dengue hospitalization costs in Colombo totaling 4.35 million USD, with per-patient costs at secondary hospitals ranging from 264 USD to 743 USD for pediatric cases, and 239 USD to 1056 USD for adults. Despite free medical care at local health centers, hospitalization costs still represent 20-35% of monthly household income, with averages of 83 USD for DF and 150 USD for DHF/dengue shock syndrome (DSS). Comparisons with other countries reveal differences in cost structures: Brazil’s public sector reported mean hospitalization costs of 770 USD for those aged <15 years, 293 USD for those aged 15-60 years, and 328 USD for those aged >60 years. [25] In Malaysia, hospitalization costs averaged 599 USD per case in the private sector and 559 USD in the public sector, while Thailand’s average cost per dengue case was 282 USD [26,27].

Indirect costs often outweigh direct non-medical costs. Shepard et al. reported indirect costs of 48 USD, compared with 10 USD in direct non-medical costs per case [17]. Fernando et al. estimated household out-of-pocket expenses averaging 80 USD per episode, rising with disease severity from 71 USD for DF to 106 USD for DHF or DSS [18]. Weerasinghe et al. reported higher household costs of 386 USD per hospitalized patient, accounting for 77.29% of monthly income, likely reflecting variations in patient demographics and access to free care [11]. Indirect costs, such as productivity losses, also contribute substantially, further amplifying the economic burden. Weerasinghe et al. estimated mean income losses of 358 USD for households with hospitalized adults in Sri Lanka, while Malaysia and Brunei reported similar figures of 337 USD and 352 USD, respectively. The study also reported average wage losses of 216 USD per adult patient due to missed workdays [11,28].

Studies also highlighted the societal impact of dengue on education and caregiving, with children in Sri Lanka missing an average of 4.6 school days and caregivers losing 4.5 workdays, as reported by Fernando et al. [18]. Comparable findings were observed in Malaysia and Thailand, where children missed 4.1 and 5.7 school days, and adults lost 7.2 and 4.6 workdays, respectively [16,27].

Another significant factor contributing to the economic burden is the substantial cost of prevention efforts, including investments in vector control initiatives. While these interventions remain essential to national dengue management, they require substantial recurring financial investment, especially during outbreaks. Evidence from dengue-endemic countries such as Thailand and Indonesia suggests that introducing dengue vaccination as an additional preventive measure can yield long-term cost savings by preventing hospitalizations, reducing severe disease, and improving productivity and quality of life (Supplementary reference 1 and 2). During the 2012 epidemic in Colombo, Thalagala et al. [12] estimated total dengue control costs at 1,704,332 USD, driven primarily by personnel expenses (79%) and consumables (16%). Such expenditures surge during outbreaks because of intensified surveillance, vector control operations, and emergency response measures. Evidence from Thailand shows that preventive vaccination with TAK-003 could avert up to 41-57% of symptomatic infections and 47-70% of hospitalizations, resulting in substantial reductions in outbreak-response spending and pressure on hospital services (Supplementary reference 1). By preventing a significant portion of cases that would otherwise escalate into emergencies, vaccination offers a strategic opportunity to avoid these high, reactive costs and support more sustainable, planned investment in Sri Lanka’s dengue control program. Liyanage et al. evaluated the CIMIC dengue vector control program, estimating a total cost of 400,496 USD, equating to 1.63 USD per person protected [20]. These findings underscore the financial strain of dengue control measures and the importance of developing cost-effective strategies.

Zeng et al. evaluated the first licensed dengue vaccine to analyze the cost-effectiveness of dengue vaccination in 10 endemic countries, including Asian nations, using a mathematical model over a 30-year horizon with 80% coverage [29]. Vaccination programs globally have demonstrated the ability to significantly reduce disease-related health care costs by preventing severe illness and limiting hospital admissions. In Sri Lanka, where dengue places a recurring strain on public health resources, an effective vaccine could provide important additional protection alongside existing vector control measures. Although the estimated annual investment in dengue vaccination by Perera et al. is approximately 28.95 million USD, this cost must be viewed in the context of the substantial expenditures associated with hospitalization, loss of income, and community disruption during outbreaks [21]. By reducing the number of severe dengue cases requiring inpatient care and minimizing productivity losses among affected households, vaccination has the potential to substantially lower the long-term economic burden of dengue. Evidence from other dengue-endemic countries supports this approach, showing that integrating vaccination with current prevention strategies can produce meaningful health and economic benefits. Therefore, the introduction of dengue vaccination in Sri Lanka could serve as a valuable, cost-saving addition to national dengue control efforts [30].

Cost-effectiveness analyses of the TAK-003 dengue vaccine conducted across multiple countries consistently demonstrate its economic viability and public health benefits. A cost-utility analysis evaluating the TAK-003 dengue vaccine in Thailand assessed routine vaccination for 11-year-olds using an assumed 87% coverage, which may not be immediately achievable at program introduction. The analysis projected that, once coverage levels stabilize in later years, TAK-003 vaccination strategies could be cost-saving, preventing an estimated 41-57% of symptomatic cases and 47-70% of hospitalizations, with societal savings of up to 1.95 billion USD and payer savings of up to 979.2 million USD over 20 years (29, Supplementary reference 1). The study concluded that vaccination was cost-saving, despite an initial vaccine cost of 628 million USD. Model analyses conducted within a public health framework similar to that of Sri Lanka show that, in Indonesia, routine vaccination with catch-up cohorts (age 9-12 years) avoided up to 57-66% of hospitalizations and remained cost-saving under all scenarios, with a 97.2% probability of being cost-effective at a willingness-to-pay threshold of 4920 USD per disability-adjusted life year (DALY) averted (Supplementary reference 2). Similarly, a study in Malaysia analyzing vaccination strategies reported up to 34-42% fewer symptomatic cases and savings of 1.06 billion USD from the societal perspective and 307 million USD from the payer perspective over 30 years (routine at age 7 plus four catch-up cohorts) (Supplementary reference 3). In Puerto Rico, modeling projections estimated that the TAK-003 vaccine could reduce symptomatic dengue by 34% and hospitalizations by 51%, with a predicted 408 million USD in savings from the societal perspective and 47 million USD from the payer perspective, and 5314 DALYs averted (Supplementary reference 4). Across these studies, TAK-003 vaccination was consistently projected to be more effective and less costly than no vaccination or alternative vaccine strategies, supporting its integration into national immunization programs in dengue-endemic countries.

Thus, while up-front costs for dengue vaccination are high, long-term reductions in direct medical costs, productivity losses, and school absenteeism make it an economically viable intervention [30].

This review also highlights several limitations. Many studies lacked data on indirect costs associated with ambulatory care, an essential aspect of the economic burden that is evident in studies from other dengue-endemic regions. Methodological differences across studies also contributed to variability in cost estimates, complicating direct comparisons. Future research using standardized methodologies could improve comparability and provide a more comprehensive understanding of dengue’s economic impact.

## Conclusion

In conclusion, dengue imposes a substantial and recurring economic burden on Sri Lanka’s public health system, particularly during epidemic years. The burden is exacerbated by high out-of-pocket expenses and missed school days, impacting households and communities. Given the annual fluctuation in dengue incidence, further studies examining costs across epidemic and non-epidemic years are essential for dynamic assessments. Such data would enable policymakers to allocate resources effectively between treatment and prevention, supporting the adoption of new technologies such as vaccines and other public health interventions. Future research using standardized and consistent methodologies will improve comparability across studies and offer a clearer, more comprehensive understanding of the economic impact of dengue.

## Declaration of competing interest

The authors have no competing interests to declare. Dr. Puneet Kalra, Dr. Vasanth Kumar, Dr. Rohan Chakraborty, and Dr. Ravdeep Kaur are employees of Takeda Biopharmaceuticals India Pvt. Ltd. Riona Hanley is an employee of Takeda Pharmaceuticals International AG, Zürich, Switzerland, and holds stock options with Takeda. Elaine Gallagher was employed by Takeda Pharmaceuticals International AG, Zürich, Switzerland during the manuscript preparation.

## Appendix Supplementary materials

Image, application 1

Image, application 2
