# Supplementary material for: Evaluating the economic burden of dengue in Sri Lanka: A systematic review of costs from 2010 to 2024
Source: IJID Reg. 2026 Jan 7;18:100837. doi: 10.1016/j.ijregi.2026.100837 (PMC12887775; doi:10.1016/j.ijregi.2026.100837)
Supplement: Supplementary file 1 [file mmc1.docx]

**PROSPERO Registration Number**

*Systematic Review Registration: PROSPERO registration number: CRD42021266800.*

**Flowchart for economic burden study**

PRISMA flowchart of the cost study selection process

Publications identified by database search (n=74)

Embase=31; PubMed=37; Cochrane=6

Keywords used: dengue, economic impact, Sri Lanka, cost of dengue, direct cost, and indirect cost

Title and abstract review (n=64)

Publications excluded because of exclusion criteria (n=55)

P=2; O=52; S=1

Full-text publication review (n=9) + 1 article known to the authors=8

Publications excluded because of exclusion criteria (n=2)

P=2

**Identification**

**Screening**

Duplicates removed (n=10)

**Eligibility**

Full-text publications included (n=8)

D, duplicates; O, outcome; P, population; S, study design
