# Supplementary material for: Evaluating the economic burden of dengue in Sri Lanka: A systematic review of costs from 2010 to 2024
Source: IJID Reg. 2026 Jan 7;18:100837. doi: 10.1016/j.ijregi.2026.100837 (PMC12887775; doi:10.1016/j.ijregi.2026.100837)
Supplement: Supplementary file 2 [file mmc2.docx]

**Supplementary References**

1. Shen J, Kharitonova E, Biswal S, Sharma M, Aballea S, Tytula A, et al. Cohort Optimization for Maximum Public Health Impact of Vaccination With TAK-003: A Case Study With Application in Thailand. Abstract presented at: 6th Asian Dengue Summit; 2023 June 15-16; Bangkok, Thailand.
2. Thobari JA, Indarti HT, Welly W, Sruamsiri R, Langer J. Assessing the Cost-Effectiveness of Dengue Vaccination with TAK-003 as Part of the National Immunization Program in Indonesia. Abstract presented at: 7th Asia Dengue Summit 2024; 2024 June 5-7; Kuala Lumpur, Malaysia.
3. Azzeri A, Jaafar H, Ting R, Langer J, Dahlui M. Potential Public Health Impact Of Dengue Vaccination With TAK-003 In Malaysia. Abstract presented at: 7th Asia Dengue Summit 2024; 2024 June 5-7; Kuala Lumpur, Malaysia.
4. Shen J, Hanley R, Kharitonova E, Janusz Z, Biswal S, Sharma M, et al. Public Health Impact and Cost-Effectiveness of a New Dengue Vaccine (TAK-003) With a Large Catch-Up Cohort in Puerto Rico. Abstract presented at: The International Society for Pharmacoeconomics and Outcomes Research (ISPOR) 2024; 2023 May 5-8; Atlanta, GA, USA.

***List of abbreviations:***

| **Abbreviations** | |
| --- | --- |
| CIMIC | Civil-Military Cooperation |
| CPI | Consumer Price Index |
| DALY | Disability-Adjusted Life Year |
| DARE | Database of Abstracts of Reviews of Effects |
| DENV | Dengue Virus |
| DEF | Data Extraction Form |
| DF | Dengue Fever |
| DHF | Dengue Hemorrhagic Fever |
| DSS | Dengue Shock Syndrome |
| GDP | Gross domestic product |
| ICU | Intensive Care Unit |
| INT$ | International Dollars |
| LKR | Sri Lanka Rupees |
| NA | Not Applicable |
| NHS | National Health Service |
| NR | Not Reported |
| PICOS | Population, Intervention, Comparator, Outcomes, and Study design |
| PRISMA | Preferred Reporting Items for Systematic Reviews and Meta-Analyses |
| RoB | Risk of Bias |
| SEARO | WHO Regional Office for South-East Asia |
| SLR | Systematic Literature review |
| USD | United States dollar |
| WHO | World Health Organization |
| WHOLIS | WHO Library Information System |
| WPSAR | Western Pacific Surveillance and Response |
| WTP | Willingness-to-pay |
